# Calcium as a Cardiac Stimulant in Pneumonia

**Published:** 1907-03-30

**Authors:** 


### CALCIUM AS A CARDIAC STIMULANT IN PNEUMONIA.

Sir Lauder Brunton was one of the first to recommend the administration of strychnine and oxygen in cases of pneumonia fifteen years ago. Since then the value of this treatment in warding off cardiac failure and supporting the patient's strength during the critical period of the disease has received continual and general confirmation. He now writes to advocate the use of calcium salts as a cardiac tonic in the same disease. He was led in the first instance to administer these salts by a consideration of the fact of their use in the physiological laboratory. Professor Ringer discovered that the contractility of the frog's heart could be maintained for some time by a perfusion of a solution of calcium salts, such as exists in ordinary tap water. It occurred to him, therefore, that they might be equally efficacious in cases of threatened cardiac failure, and he is considerably impressed with their therapeutic value in these conditions. He recommends calcium chloride in 5 to 10 grain doses every four hours, simply dissolved in water. The unpleasant taste is best covered by a little saccharine. One minim of the elixir of saccharine is sufficient to cover the taste of 10 grains of the salt.

